# Comparison of Levels of Three Tobacco Smoke Exposure Biomarkers in Children of Smokers

**DOI:** 10.3390/ijerph182211803

**Published:** 2021-11-10

**Authors:** E. Melinda Mahabee-Gittens, Georg E. Matt, Lili Ding, Ashley L. Merianos

**Affiliations:** 1 Division of Emergency Medicine, Cincinnati Children’s Hospital Medical Center, University of Cincinnati College of Medicine, Cincinnati, OH 45229, USA; 2 Department of Psychology, San Diego State University, San Diego, CA 92123, USA; gmatt@sdsu.edu; 3 Division of Biostatistics and Epidemiology, Cincinnati Children’s Hospital Medical Center, University of Cincinnati College of Medicine, Cincinnati, OH 45229, USA; lili.Ding@cchmc.org; 4 School of Human Services, University of Cincinnati, Cincinnati, OH 45221, USA; ashley.merianos@uc.edu

**Keywords:** children, biomarkers, tobacco smoke exposure, secondhand smoke, thirdhand smoke

## Abstract

**Objectives**: Cotinine, 4-(methylnitrosamino)-1-(3-pyridyl)-1-butanol (NNAL), and N-oxides are biomarkers of tobacco smoke exposure (TSE) used to assess short- and longer-term TSE. The objective of this study was to assess the associations between these TSE biomarkers, sociodemographics, parental smoking, and child TSE patterns among 0–17-year-olds. **Methods**: A convenience sample of 179 pediatric patients (mean (SD) age = 7.9 (4.3) years) who lived with ≥1 smoker and who had parental assessments completed and urine samples analyzed for the three TSE biomarkers of interest were included. Biomarker levels were log-transformed, univariate regression models were built and Pearson correlations were assessed. **Results**: In total, 100% of children had detectable levels of cotinine and >96% had detectable NNAL and N-oxide levels. The geometric means of cotinine, NNAL, and N-oxide levels were 10.1 ng/mL, 25.3 pg/mL, and 22.9 pg/mL, respectively. The mean (SD) number of daily cigarettes smoked by parents was 10.6 (6.0) cigarettes. Child age negatively correlated with urinary cotinine (*r* = −0.202, *p* = 0.007) and log NNAL levels (*r* = −0.275, *p* < 0.001). The highest log-cotinine levels were in children who were younger, of African American race, and whose parents had a lower education, an annual income ≤USD15,000, and no smoking bans. The highest log-NNAL and N-oxide levels were in children whose parents had a lower education, had no smoking bans, and were around higher numbers of cigarettes. **Conclusion**: Children of smokers who were younger, African American, and had no smoking bans had the highest TSE biomarker levels. Targeted interventions are needed to reduce TSE levels among high-risk children.

## 1. Introduction

Despite recent decreases in adult tobacco product use [1], tobacco smoke exposure (TSE) remains prevalent in U.S. children. Examination of children in the 2017–2018 National Health and Nutrition Examination Survey (NHANES) cohorts indicated that 38% of 3–11-year-olds and 33% of adolescents have biochemically confirmed TSE [2]. When children are exposed to tobacco smoke, they can be acutely exposed to secondhand smoke (SHS) and/or chronically exposed to thirdhand smoke (THS), which is aged tobacco smoke residue that lingers in dust and on surfaces in indoor environments where tobacco was used previously [3]. While exposure to SHS occurs during and up to a few hours after active smoking takes place, exposure to THS can occur for days or years after tobacco products have been burned [3]. The measurement of multiple TSE biomarkers can provide insight into variations in TSE patterns in children as exposure may be brief, over short periods of time (e.g., while visiting a relative’s house), intermittent (e.g., every weekend), or over longer periods of time (e.g., while living in homes without smoking bans).

The characteristics of biomarkers that make them useful for TSE research include their ability to accurately estimate low and high levels of TSE, demonstrate a dose–response relationship between reported TSE levels and the biomarker, be detectable in biological samples that are easy to collect (e.g., urine, saliva), be detectable using reproducible laboratory methods, and remain stable upon storage for future analyses [4,5,6]. Cotinine is the most commonly measured and reported TSE biomarker that can be assessed in the urine, saliva, blood, hair, or nails of exposed children [5]. Cotinine is a metabolite of nicotine and measures recent TSE given its average half-life of 16–28 h [7,8,9]. Although cotinine has excellent sensitivity for recent TSE, cotinine levels can be affected by other factors including age, sex, and genetic variation in the frequency of CYP2A6 variant alleles among different racial/ethnic groups [5,9,10,11,12,13]. Several studies have demonstrated that sociodemographic characteristics and TSE patterns associated with higher cotinine levels in children are young age; non-Hispanic, African American race; poverty; residing in rented homes or homes without smoking bans; and being around more smoked cigarettes [14,15,16,17,18].

Cotinine levels provide information about children’s exposure to nicotine that occurs 2–4 days prior to measurement [19]. In order to assess short- and long-term TSE, there are two additional tobacco-specific biomarkers that are highly detectable in urine after an individual has been exposed. The first is 4-(methylnitrosamino)-1-(3)pyridyl-1-butanol (i.e., NNAL), which is a metabolite of 4-(methylnitrosamino)-1-(3)pyridyl-1-butanone (i.e., NNK), a potent lung carcinogen [20,21]. NNAL has a half-life of 10–40 days; thus, it measures long-term or intermittent TSE that has occurred over the past month or longer [19]. Compared to nicotine metabolites such as cotinine, NNAL may be a more suitable biomarker for chronic THS exposure [22]. NNAL levels are up to three times higher in children with TSE compared to adults with TSE [23], with the highest levels observed in children who are African American, live in homes without smoking bans, and are around more cigarettes [24,25,26].

N-oxides are metabolites of nicotelline and tobacco-specific markers for particulate matter derived from tobacco smoke [27]. Nicotelline is present almost entirely in the particulate phase of SHS and THS; thus, N-oxides are environmental markers of particulate matter derived from tobacco smoke [27]. N-oxides have a half-life of approximately two hours [27], and thus levels reflect very recent TSE. To the best of our knowledge, it appears that N-oxides have not been studied in children. In this study, we measured levels of urinary cotinine, urinary NNAL, and urinary N-oxides to assess the range of recently and longer-term exposure in children who lived with smokers and thus had known TSE. The specific study objectives were to examine and compare levels of urinary cotinine, NNAL, and N-oxides and associated sociodemographics, parental smoking patterns, and child TSE patterns among 0–17-year-old nonsmoking children who lived with smokers.

## 2. Methods

### 2.1. Study Design

Participants were 0–17-year-old children who were being treated in a pediatric emergency department (PED) or urgent care (UC) in a children’s hospital in the U.S. between the time period of April 2016 to May 2019. Children were accompanied to one of these sites by their parents/legal guardians. Child–parent dyads were enrolled in a 2-group, randomized controlled trial of a parental tobacco cessation intervention called “Healthy Families” (www.clinicaltrials.gov: NCT02531594 accessed on 8 September 2021). All parents in the Healthy Families trial were active, daily smokers and the children were nonsmokers. The intervention was designed to help parents stop smoking and reduce children’s TSE. Further details on intervention components are available elsewhere [28]. The hospital’s institutional review board approved this study; parental consent for all children and child assent for children ≥11 years old were obtained prior to conducting the study.

Children were eligible if they were age 0–17 years old, had a potential TSE-related chief complaint (e.g., wheezing), lived with a parent who smoked cigarettes daily, denied the use of any tobacco products (e.g., cigars, electronic cigarettes) or cannabis products, and had urine samples collected during their PED/UC visit. A total of 179 participants met these eligibility criteria and had biomarker data of interest available for analysis. We analyzed data and samples from the PED/UC visit, which occurred prior to any cessation intervention activities.

### 2.2. Questionnaires

Parents completed electronic assessments during their child’s PED/UC visit, which included questions on sociodemographics: child age, sex, race, ethnicity, insurance type; parental highest education level (i.e., ≤high school graduate/equivalent, which included those who attended high school but did not graduate or those who graduated from high school and ≥some college, which included those who attended college but did not graduate or those who graduated from college); annual household income, and housing type (e.g., single-family, multiunit housing (MUH) such as townhouse or apartment building). We also obtained the child’s height and weight from the child’s electronic medical record to calculate age- and sex-specific body mass index z-scores (BMIZ) based on the Centers for Disease Control and Prevention 2000 growth charts [29].

Parents self-reported their smoking patterns: (1) the number of cigarettes they smoked per day and (2) their current daily electronic cigarette (e-cigarette) use. Parents also self-reported their child’s TSE patterns: (1) home and car smoking rules—these questions were added after the study started and only *n* = 111 were asked these questions; participants who reported that they never allowed smoking in the home and car were classified as having a comprehensive smoking ban, (2) cumulative number of household smokers, and (3) cumulative child TSE. The latter was calculated by totaling the daily number of cigarettes smoked around the child by all smokers (e.g., mother, father, siblings, visitors, relatives) in any location (e.g., home, car) in the past week.

### 2.3. Urine Sample Collection and Analysis

Urine samples were collected from 179 patients, immediately frozen at −80 °C and shipped on dry ice to the analyzing laboratory (Jacob Lab, University of California at San Francisco), at which they were stored and frozen at −20 °C until analysis for cotinine (*n* = 177), NNAL (*n* = 179), and N-oxides (*n* = 176) with liquid chromatography–tandem mass spectrometry (LC–MS/MS) [27,30,31]; limit of quantitation (LOQ) was: 0.02 ng/mL for cotinine, 0.25 pg/mL for NNAL, and 1 pg/mL for N-oxides. All participants with measured cotinine had detectable levels, 99.4% of children had detectable NNAL levels, and 96.6% of children had detectable N-oxides levels. Values below the LOQ were imputed as LOQ/√2 for analysis.

### 2.4. Statistical Analysis

Biomarker levels were log-transformed to address skewed distributions prior to analyses, and we report geometric means (GeoMs), 95% confidence intervals (95% CIs), medians (Mdns), and interquartile ranges (IQRs). In separate univariate regression models, we assessed individual associations of child sociodemographics and parent-reported smoking and child TSE patterns with urinary cotinine, NNAL, and N-oxide outcome measures. We determined Pearson correlations to describe the strength of bivariate linear correlations between the biomarker and quantitative child sociodemographics (i.e., child age and child BMIZ) and parent-reported smoking and child TSE patterns (i.e., number of cigarettes smoked per day by parents, number of cigarette smokers per day around the child in any location, and cigarettes per day smoked around the child by all smokers in any location). Statistical analyses were conducting using SAS version 9.4, and the Type I error was 0.05 (two-tailed).

## 3. Results

### 3.1. Child Sociodemographics

The mean (SD) child age was 7.9 (4.3) years. As seen in Table 1, the majority of children were male (54.2%), African American (68.2%), of non-Hispanic origin (97.8%), and had public insurance or were self-paying (92.7%). Over half of children had parents with an education level ≥some college (51.4%), and the majority had an income level ≤USD15,000 (69.1%) and lived in multi-unit housing or apartment buildings (62.0%). The average (SD) child BMIZ was 0.8 (1.2).

### 3.2. Parental Smoking and Child TSE Patterns

All children (100%) lived with at least one smoker. As seen in Table 2, the median number of cigarettes smoked per day by parents was 10.0 (IQR = 6.0–15.0). Only 5% of parents reported current e-cigarette use. Regarding implemented smoking bans, of the 111 children’s parents asked these questions, 32.4% of children had a home smoking ban, 36.9% had a car smoking ban, and 15.3% had a comprehensive smoking ban (i.e., a home and car smoking ban). Children were around a median of two (IQR = 1.0–3.0) cigarette smokers per day in any location, and children were around a median of five (IQR = 0.0–10.0) cigarettes per day from all smokers in any location. In total, 26.8% of children were reportedly around 0 cigarettes per day.

### 3.3. Urinary Cotinine, NNAL, and N-oxides Levels

All 177 children who had urine samples that were analyzed for cotinine had detectable urinary cotinine levels which ranged from 0.14 to 169.01 ng/mL (GeoM = 10.13 ng/mL; 95% CI = [8.33; 12.32], Mdn = 11.64 ng/mL, IQR = 4.66–26.55). Of the 179 children who had urine samples that were analyzed for NNAL, 178 (99.4%) had detectable levels, which ranged from 0.18 to 489.26 pg/mL (GeoM = 25.32 pg/mL; 95% CI = [20.97; 30.57], Mdn = 30.88 pg/mL, IQR = 12.40–60.47). Of the 176 children who had urine samples that were analyzed for N-oxides, 170 (96.6%) had detectable levels which ranged from 0.97 to 371.65 pg/mL (GeoM = 22.94 pg/mL; 95% CI = [18.75; 28.08], Mdn = 26.52 pg/mL, IQR = 9.19–67.97).

Urinary cotinine was moderately and positively correlated with NNAL (*r* = 0.40, *p* < 0.001) and N-oxides (*r* = 0.46, *p* < 0.001). NNAL and N-oxides also showed a moderate positive correlation (*r* = 0.49, *p* < 0.001).

### 3.4. Bivariate Associations between Sociodemographics and Urinary Cotinine Levels, NNAL Levels, and N-oxide Levels

As seen in Table 1, child age negatively correlated with urinary cotinine (*r* = −0.202, *p* = 0.007). Simple linear regression results revealed a negative relationship between child age and urinary cotinine, with 0–1-year-olds (GeoM = 27.7 ng/mL) having the highest levels when compared to 2–4-year-olds (GeoM = 11.1 ng/mL, *p* = 0.044), 5–9-year-olds (GeoM = 9.6 ng/mL, *p* = 0.009), and 10–17-year-olds (GeoM = 8.5 ng/mL, *p* = 0.005). African American children (GeoM = 12.5 ng/mL, *p* < 0.001) had significantly higher mean cotinine compared to White children (GeoM = 5.0 ng/mL). Children with significantly greater mean cotinine levels had parents with ≤high school graduate/equivalent education (GeoM = 13.4 ng/mL, *p* = 0.005) and had a household income ≤USD15,000 (GeoM = 12.5 ng/mL, *p* = 0.001) compared to children with parents who had ≥some college (GeoM = 7.8 ng/mL) and had a household income level >USD15,000 (GeoM = 6.2 ng/mL).

Child age also negatively correlated with NNAL (*r* = −0.275, *p* < 0.001) levels, showing higher levels among younger children. Children had significantly greater mean NNAL levels if they had parents with ≤high school graduate/equivalent education (GeoM = 31.6 pg/mL, *p* = 0.024) compared to children with parents who had ≥some college (GeoM = 20.5 pg/mL).

No associations were found between child age and N-oxide levels. Children with significantly higher mean N-oxide levels were more likely to have parents with ≤high school graduate/equivalent education (GeoM = 28.7 pg/mL, *p* = 0.033) than children with parents who had ≥some college (GeoM = 18.6 pg/mL).

### 3.5. Bivariate Associations between Parental Smoking and Child TSE Patterns and Urinary Cotinine Levels, NNAL Levels, and N-oxide Levels

As seen in Table 2, children with a home smoking ban implemented (GeoM = 6.6 ng/mL, *p* = 0.004) or a comprehensive smoking ban implemented (GeoM = 5.2 ng/mL, *p* = 0.009) had lower mean urinary cotinine levels compared to children without a home or comprehensive smoking ban implemented (GeoM = 14.2 ng/mL and GeoM = 12.8 ng/mL, respectively). 

Children with a home smoking ban implemented (GeoM = 16.6 pg/mL, *p* = 0.014) or a comprehensive smoking ban implemented (GeoM = 14.4 pg/mL, *p* = 0.047) also had lower mean urinary NNAL levels compared to children without a home or comprehensive smoking ban implemented (GeoM = 32.2 pg/mL and GeoM = 28.9 pg/mL, respectively). Children who were around 6–14 cigarettes per day (GeoM = 34.9 pg/mL, *p* = 0.004) had significantly higher mean NNAL levels than children around 0 cigarettes per day (GeoM = 16.6 pg/mL).

There was an increase in N-oxide levels as children were exposed to more cigarettes (1–5 cigarettes/day: GeoM = 14.9 pg/mL; 6–14 cigarettes/day: GeoM = 26.7 pg/mL; 15–40 cigarettes/day: GeoM = 28.4 pg/mL). Children from homes with a home smoking ban had lower NNAL levels (GeoM = 14.4 pg/mL, *p* = 0.004) than children without a home smoking ban (GeoM = 31.7 pg/mL).

## 4. Discussion

This is the first study to examine levels of urinary cotinine, urinary NNAL, and urinary N-oxides in racially diverse children who lived with smokers. All children had detectable cotinine levels and over 96% of children had detectable levels of NNAL and N-oxides. Our results add to the literature by including measurements of NNAL and N-oxides, which are tobacco-specific biomarkers with short-term and longer-term half-lives. N-oxides detect very recent TSE (over a few hours) [27], cotinine detects recent TSE (i.e., over a few days) [7,8,9], and NNAL detects intermittent or longer-term exposure of up to 6–12 weeks after exposure [19]. Compared to other studies of child nonsmokers with TSE, the median levels of cotinine (11.6 ng/mL in this study compared to median cotinine levels of 0.3–1.4 ng/mL in other studies [24,26,32]) and the median levels of carcinogenic biomarker uptake of NNAL (30.9 pg/mL in this study compared to median NNAL levels of 0.4–2.2 pg/mL in other studies [24,26,32]) that we observed were up to 39 and 77 times higher, respectively. These high differences may have been due to the higher overall TSE in our study sample compared to previous cohorts. N-oxide levels have not been previously measured in children; however, the levels observed in this study ranged from 0.97 to 371.65 pg/mL (GeoM = 22.94 pg/mL), in comparison to N-oxide levels that ranged from 0 to 141 pg/mL (mean (SD) 32.8 (55.0) pg/mL) in a subset of current adult electronic cigarette users and current dual combustible cigarette and electronic cigarette users [33]. These findings indicate that by using multiple TSE biomarkers which have different half-lives and which measure different tobacco smoke pollutants, we were able to capture a wider range of children’s TSE than would have been possible if cotinine alone were measured. These findings also indicate that children who live with smokers have high levels of both SHS and THS exposure, which is a risk factor for adverse health effects [14,17,34,35]. Additionally, the potential acute and long-term clinical effects of these findings are concerning given research that indicates that NNAL may be a biomarker of future cancer risk in children [14,36,37,38] and because the acute and long-term clinical risks associated with N-oxide levels are currently unknown. 

Similar to prior research [16,35], we observed a negative correlation between child age and urinary cotinine, but a new finding was that there was a negative correlation between child age and NNAL levels, with the highest levels in 0–1-year-old children. This association may be due to increased TSE in young children, who spend more time at home and are thus are exposed to more home tobacco smoke pollutants [39]. The lack of a correlation with child age and N-oxides may be due to the short half-life of N-oxides of only 2–3 h in comparison to the longer half-lives of cotinine and NNAL. This short half-life combined with the long wait times that children in this study had to be seen in the PED/UC and subsequently enrolled in this study [27] may have contributed to this result. In parallel to other research [18,26], we found that cotinine levels were higher in children who were African American, had lower household incomes, had parents with a high school education or lower, and had no home smoking bans. Adding to the extant literature, we found that NNAL and N-oxide levels were highest in children: whose parents had a high school education or lower, who did not have home smoking bans, and who were around more cigarettes in any location. Notably, race was associated with cotinine levels but not with NNAL or N-oxide levels. This may be because there is genetic variation in the cytochrome P450 (CYP) 2A6 variant alleles which catalyzes the metabolism of cotinine from nicotine [9,11,12], but it is unknown if these alleles affect NNAL and N-oxide levels in children with TSE. However, other research has found that NNAL levels are higher in children who are African American [24,25]; thus, these results need to be examined further.

We found a small to medium positive correlation (*r* = 0.4–0.5) between urinary cotinine and NNAL and between cotinine and N-oxide levels; other research has also found correlations between cotinine and NNAL [23,24,38,40]. These correlations indicate that these biomarkers account for 16% to 25% of their variance and that 60–80% of the variance is unique to very recent exposure (i.e., past 2 h for N-oxides, which could be equivalent to wait times in the PED/UC setting), the past 1–3 days (i.e., as assessed with cotinine), and the past 30–40 days (as assessed with NNAL). Since NNAL, cotinine, and N-oxides capture different aspects and timing of children’s exposure to tobacco smoke pollutants, these findings suggest that a more comprehensive assessment of TSE using multiple biomarkers is needed in order to study the impact of different aspects of exposure patterns on acute and especially longer-term health outcomes.

This study had several strengths, including the measurement of NNAL and N-oxides in addition to the commonly measured biomarker cotinine, which provides a range of timing of exposure to tobacco smoke. Collectively, the results of this study indicate that SHS and THS exposure as detected with biomarkers of shorter- and longer-term exposure are high in the children of smokers. Despite these strengths, there were also limitations, including the use of a convenience sample of children who were seen at a PED/UC site that was part of one children’s hospital, and the cross-sectional nature of the study. Thus, our findings cannot be generalized to other settings or populations and causal associations cannot be determined. Parent reports of their smoking patterns and their child’s TSE patterns were subject to reporting and/or recall bias, but a strength is that TSE was biochemically confirmed with three biomarkers. Furthermore, since all parents were not asked about home and car smoking bans, conclusions cannot be drawn about smoking bans and TSE biomarker levels. We did not assess the use of heated tobacco products, nor did we analyze whether biomarker levels differed by tobacco product type (e.g., cigarettes vs. cigars). Finally, we did not correct the NNAL levels by urine creatinine or specific gravity, which may have affected our findings [41]. Future studies should consider examining these TSE biomarkers in larger samples of children who live with smokers and nonsmokers so that levels and associated characteristics can be compared. There is a need for rigorous longitudinal studies to better ascertain the patterns of these biomarkers and the associated clinical effects and risks over time. 

## 5. Conclusions

In conclusion, this study adds to the literature on TSE in children as it underscores the importance of measuring multiple biomarkers which may assess both SHS and THS exposure, since no single biomarker captures the diversity of recent, intermediate, and long-term exposure patterns. Children who are young, racially diverse, and those who live in homes with no smoking bans are at increased risk of exposure. Given the knowledge that TSE in children can lead to future morbidity, targeted interventions are urgently needed to decrease both SHS and THS exposure in at-risk children. In the clinical setting, these interventions should include screening all parents for current tobacco use and providing brief counseling to parents about the dangers of SHS and THS on children’s current and future health and the benefits of quitting tobacco product use for both their children’s and their own health. TSE screening and brief interventions can take less than three minutes, are well-received by parents and staff, and can be conducted with limited interruptions to the pediatric clinical workflow [42,43].

## Figures and Tables

**Table 1:** Sociodemographics by Urinary Cotinine, NNAL, and N-oxides Levels of Pediatric Emergency Department and Urgent Care Patients.

		Cotinine Concentration (ng/mL) (*n* = 177)			NNAL Concentration (pg/mL) (*n* = 179)			N-oxides Concentration (pg/mL) (*n* = 176)		
Characteristic	Overall *n* (%) ^a^	GeoM (95% CI)	Mdn (IQR)	*p*-Value ^b^	GeoM (95% CI)	Mdn (IQR)	*p*-Value ^b^	GeoM (95% CI)	Mdn (IQR)	*p*-Value ^b^
**Child Age, Mean (SD)**	7.9 (4.3)	*r* = −0.202	-	**0.007**	*r* = −0.275	-	**<0.001**	*r* = −0.132	-	0.080
**0–1 years**	12 (6.7)	27.7 (12.9–59.4)	24.2 (13.6–78.4)	Ref	29.8 (10.9–81.2)	46.9 (10.5–97.2)	Ref	29.5 (13.1–66.2)	25.2 (9.0–107.7)	Ref
**2–4 years**	29 (16.2)	11.1 (6.9–18.0)	13.6 (6.5–28.2)	**0.044**	47.2 (28.5–78.2)	48.8 (27.9–128.0)	0.282	30.7 (18.7–50.4)	53.3 (12.8–86.0)	0.934
**5–9 years**	77 (43.0)	9.6 (7.4–12.4)	10.3 (4.5–21.4)	**0.009**	26.5 (20.7–33.9)	33.7 (13.3–57.7)	0.760	21.4 (15.8–29.0)	23.9 (7.5–56.7)	0.449
**10–17 years**	61 (34.1)	8.5 (5.7–12.6)	12.0 (3.3–27.5)	**0.005**	17.2 (17.2–12.3)	23.2 (9.4–40.8)	0.163	20.8 (14.3–30.3)	26.4 (8.9–65.4)	0.420
**Child Sex**										
**Male**	97 (54.2)	11.0 (8.4–14.5)	13.3 (5.0–28.1)	Ref	28.6 (21.8–37.5)	35.0 (14.1–72.7)	Ref	24.8 (18.9–32.4)	32.6 (9.8–70.0)	Ref
**Female**	82 (45.8)	9.2 (6.9–12.2)	11.1 (4.4–24.0)	0.355	22.0 (16.9–28.5)	23.6 (10.2–55.1)	0.170	21.0 (15.4–28.6)	23.0 (7.9–60.3)	0.417
**Child Race**										
**White**	41 (22.9)	5.0 (3.1–8.0)	5.8 (2.1–16.5)	Ref	18.1 (10.0–31.0)	21.1 (6.9–61.6)	Ref	20.2 (12.1–33.8)	26.3 (5.5–73.8)	Ref
**African American**	122 (68.2)	12.5 (10.1–15.5)	12.3 (6.4–30.4)	**<0.001**	28.3 (23.4–34.2)	33.6 (14.2–56.7)	0.054	24.0 (19.2–29.9)	26.0 (10.8–64.0)	0.501
**Other**	13 (7.3)	10.2 (4.2–25.1)	11.8 (8.6–28.3)	0.080	21.7 (8.6–55.0)	20.0 (8.0–55.1)	0.656	20.7 (6.7–63.8)	41.6 (4.5–62.6)	0.955
**Unknown**	3 (1.7)	22.3 (2.0–245.0)	25.3 (8.1–54.7)	0.051	53.2 (2.3–1210.4)	93.4 (12.6–128.0)	0.158	33.6 (1.3–870.7)	70.2 (7.4–72.9)	0.538
**Child Insurance Type**										
**Commercial**	13 (7.3)	9.1 (3.9–21.4)	11.4 (3.8–19.4)	Ref	21.0 (11.5–38.5)	20.0 (9.4–38.7)	Ref	20.9 (7.9–54.8)	18.5 (6.1–46.5)	Ref
**Public/self-paying**	166 (92.7)	10.2 (8.3–12.5)	11.6 (4.7–27.1)	0.769	25.7 (21.1–31.3)	32.1 (12.6–61.0)	0.589	23.1 (18.8–28.5)	26.7 (10.0–68.5)	0.793
**Parent Education Level**										
**≤High school graduate/equivalent**	87 (48.6)	13.4 (10.7–16.9)	13.1 (6.6–31.4)	Ref	31.6 (25.4–39.3)	33.7 (15.8–69.8)	Ref	28.7 (22.5–36.7)	29.5 (13.5–71.3)	Ref
**≥Some college**	92 (51.4)	7.8 (5.7–10.5)	10.3 (3.0–24.0)	**0.005**	20.5 (15.2–27.7)	28.8 (8.1–55.1)	**0.024**	18.6 (13.6–25.5)	22.8 (6.6–62.6)	**0.033**
**Income Level**										
**≤USD15,000**	123 (69.1)	12.5 (10.2–15.4)	13.5 (6.3–30.4)	Ref	28.8 (23.5–35.3)	35.0 (14.2–63.0)	Ref	25.0 (19.8–31.6)	26.5 (11.0–68.7)	Ref
**>USD15,000**	55 (30.9)	6.2 (4.1–9.5)	8.0 (2.4–21.7)	**0.001**	19.4 (12.8–29.2)	20.0 (8.5–48.8)	0.056	19.4 (13.0–28.9)	28.7 (6.4–68.5)	0.247
**Housing Type**										
**Single-Family**	68 (38.0)	8.5 (6.1–11.8)	11.1 (3.3–23.5)	Ref	23.1 (17.2–30.9)	23.2 (12.3–47.0)	Ref	21.8 (15.8–30.2)	21.8 (9.5–71.3)	Ref
**Multifamily or Apartment**	111 (62.0)	11.3 (8.8–14.4)	11.8 (6.1–28.3)	0.170	26.8 (20.9–34.4)	35.0 (12.4–61.6)	0.448	23.7 (18.2–30.8)	32.1 (9.0–66.2)	0.704

Abbreviations: GeoM, geometric mean; CI, confidence interval; Mdn, median; IQR, interquartile range; Ref, reference group. ^a^ *n* (%) unless otherwise noted. ^b^ *p*-values are unadjusted and refer to simple linear regression model results with one sociodemographic characteristic as the explanatory variable and the biomarkers in log scale as the response variable in each model unless noted otherwise. Bold print indicates statistical significance at *p* < 0.05. Please note: The correlation between urinary cotinine and NNAL was *r* = 0.40 (*p* < 0.001), the correlation between urinary cotinine and N-oxides was *r* = 0.46 *(p* < 0.001). The correlation between urinary NNAL and N-oxides was *r* = 0.49 (*p* < 0.001).

**Table 2:** Parent-Reported Smoking Behavior and Child TSE Patterns by Urinary Cotinine, NNAL, and N-oxide Levels of Pediatric Emergency Department and Urgent Care Patients.

		Cotinine Concentration (ng/mL) (*n* = 177)			NNAL Concentration (pg/mL) (*n* = 179)			N-oxides Concentration (pg/mL) (*n* = 176)		
Characteristic	Overall *n* (%) ^a^	GeoM (95% CI)	Mdn (IQR)	*p*-Value ^b^	GeoM (95% CI)	Mdn (IQR)	*p*-Value ^b^	GeoM (95% CI)	Mdn (IQR)	*p*-Value ^b^
**Parent No. of Cigarettes/Day, Mean (SD)**	10.6 (6.0)	*r* = 0.030	-	0.706	*r* = 0.048	-	0.549	*r* = 0.127	-	0.109
1–5 cigarettes	30 (18.6)	8.3 (5.1–13.5)	9.3 (3.7–21.4)	Ref	19.2 (11.8–31.4)	29.5 (11.4–40.8)	Ref	14.9 (8.9–24.7)	16.6 (6.4–36.3)	Ref
6–14 cigarettes	86 (53.4)	11.8 (9.0–15.5)	12.2 (6.9–29.9)	0.193	27.9 (21.7–35.9)	32.6 (14.1–61.6)	0.172	26.7 (20.2–35.4)	31.6 (12.6–71.2)	**0.043**
15–40 cigarettes	45 (28.0)	10.5 (7.0–15.7)	13.6 (4.5–26.5)	0.439	27.3 (17.7–42.1)	35.0 (13.4–66.0)	0.247	28.4 (18.7–43.1)	46.0 (10.0–75.5)	**0.043**
**Parent Current E-Cigarette Use**										
No	170 (95.0)	10.1 (8.2–12.3)	11.5 (4.4–28.0)	Ref	25.3 (20.7–30.7)	31.3 (11.9–61.0)	Ref	22.5 (18.2–27.7)	25.7 (8.5–68.5)	Ref
Yes	9 (5.0)	11.7 (4.6–29.7)	17.2 (15.3–22.4)	0.736	26.7 (15.0–47.3)	27.2 (23.2–37.7)	0.901	33.6 (16.3–69.1)	39.4 (17.4–65.4)	0.390
**Home Smoking Ban (*n* = 111)**										
No	75 (67.6)	14.2 (11.3–18.0)	14.0 (7.1–30.4)	Ref	32.2 (25.0–41.4)	36.1 (14.9–67.3)	Ref	31.7 (23.0–42.0)	32.7 (15.9–75.5)	Ref
Yes	36 (32.4)	6.6 (3.6–12.0)	8.1 (1.6–31.3)	**0.004**	16.6 (9.4–29.1)	21.9 (5.3–48.0)	**0.014**	14.4 (8.6–24.3)	9.9 (5.5–69.8)	**0.004**
**Car Smoking Ban** **(*n* = 111)**										
No	70 (63.1)	10.9 (8.0–14.8)	13.0 (4.7–28.0)	Ref	27.5 (19.6–38.5)	35.6 (11.9–67.3)	Ref	27.2 (19.5–37.9)	31.6 (10.9–72.9)	Ref
Yes	41 (36.9)	11.6 (7.3–18.4)	13.2 (5.8–35.5)	0.824	23.5 (16.1–34.5)	29.0 (8.5–54.3)	0.556	20.3 (13.2–31.3)	20.6 (7.3–66.1)	0.286
**Comprehensive Home and Car Smoking Ban ^c^** **(*n* = 111)**										
No	94 (84.7)	12.8 (10.0–16.4)	13.9 (6.9–31.3)	Ref	28.9 (22.1–37.8)	35.0 (12.7–67.3)	Ref	27.3 (20.8–35.8)	29.6 (11.0–74.2)	Ref
Yes	17 (15.3)	5.2 (2.0–13.2)	3.3 (1.6–11.5)	**0.009**	14.4 (7.0–29.2)	8.5 (4.8–38.7)	**0.047**	13.5 (6.2–29.5)	10.0 (4.6–49.1)	0.051
**Cumulative Number of Household Smokers ^d^** **Mean (SD)**	2.2 (1.3)	*r* = 0.116	-	0.127	*r* = 0.103	-	0.173	*r* = 0.057	-	0.455
1 smoker	59 (33.7)	9.4 (6.7–13.2)	11.6 (5.3–24.0)	Ref	24.9 (17.8–34.8)	33.7 (11.9–61.0)	Ref	22.7 (16.4–31.6)	31.0 (10.7–60.3)	Ref
2 smokers	56 (32.0)	9.2 (6.5–13.1)	11.2 (4.1–24.1)	0.957	26.6 (19.4–36.6)	30.3 (13.4–52.0)	0.779	26.3 (17.6–39.4)	27.6 (7.7–95.7)	0.572
3–7 smokers	60 (34.3)	12.2 (8.6–17.4)	13.2 (6.3–32.3)	0.271	24.7 (17.4–35.0)	25.9 (13.3–66.2)	0.968	21.2 (15.0–30.0)	19.0 (9.5–71.3)	0.783
**Cumulative Child TSE ^e^ Mean (SD)**	8.9 (19.6)	*r* = 0.117	-	0.134	*r* = 0.082	-	0.290	*r* = 0.077	-	0.323
0 cigarettes	45 (26.8)	9.2 (5.7–14.7)	11.3 (3.5–26.9)	Ref	16.6 (11.3–24.3)	24.2 (7.0–40.8)	Ref	15.9 (10.6–23.9)	19.6 (6.3–49.1)	Ref
1–5 cigarettes	46 (27.4)	8.3 (5.3–13.1)	11.4 (3.3–25.3)	0.721	23.9 (15.2–37.7)	31.4 (11.3–79.3)	0.164	20.2 (12.8–31.7)	22.8 (9.5–73.6)	0.399
6–14 cigarettes	51 (30.4)	11.3 (8.6–14.9)	11.6 (6.9–24.3)	0.436	34.9 (26.0–46.9)	37.7 (16.4–66.0)	**0.004**	35.3 (25.6–48.9)	43.9 (16.6–68.5)	**0.004**
15–224 cigarettes	26 (15.5)	17.2 (12.0–24.7)	22.4 (9.0–33.2)	0.054	33.8 (22.9–49.9)	31.2 (20.0–57.7)	**0.0217**	27.6 (17.0–45.0)	33.1 (12.0–83.6)	0.094

Abbreviations: GeoM, geometric mean; CI, confidence interval; Mdn, median; IQR, interquartile range; Ref, reference group; TSE, tobacco smoke exposure. ^a^ *n* (%) unless otherwise noted. ^b^ *p*-values are unadjusted and refer to simple linear regression model results with one sociodemographic characteristic as the explanatory variable and the biomarkers in log scale as the response variable in each model unless noted otherwise. Bold print indicates statistical significance at *p* < 0.05. ^c^ Comprehensive Home and Car Smoking Ban: Smoking is never allowed in the home or car. ^d^ Cumulative Number of Household Smokers: Total number of cigarette smokers/day around the child in any location. ^e^ Cumulative Child TSE: Total number of cigarettes smoked around the child daily by all smokers in any location.3.3. Urinary Cotinine, NNAL, and N-oxides Levels.

## Data Availability

The dataset will be made available to other researchers upon request for the purpose of reproducing the findings.
